# The role of external factors in shaping the supply and demand of general practitioners in Iran: a qualitative study

**DOI:** 10.1186/s12875-025-03123-2

**Published:** 2025-11-26

**Authors:** Mohammadreza Amiresmaili, Mohammad Hossein Mehrolhassani, Mahla Iranmanesh

**Affiliations:** 1 Faculty of Management and Medical Information Sciences, Kerman University of Medical Sciences, Kerman, Iran; 2 Health Services Management Research Center, Institute for Futures Studies in Health, Kerman University of Medical Sciences, Kerman, Iran; 3 Social Determinants of Health Research Center, Institute for Futures Studies in Health, Kerman University of Medical Sciences, Kerman, Iran

**Keywords:** General practitioners, Workforce, Supply and demand, Extra-system drivers, Iran, Qualitative study

## Abstract

General practitioners are crucial for community health, but their numbers and distribution in Iran face challenges due to factors outside the healthcare system. In Iran—as in many health systems experiencing epidemiologic transition, fiscal constraints, and specialist-oriented care—primary care capacity is a key determinant of equity, access, and cost containment. Yet, evidence on how extra-system (external) forces shape the GP labour market remains fragmented, limiting actionable workforce planning. This study addresses that gap by synthesising a systematic framework of external drivers to inform policy design, prioritisation, and system dynamics modelling of GP supply–demand management.

A qualitative approach using conventional content analysis was employed. Twenty-nine key individuals and experts from Iran’s health system, medical universities, the Ministry of Health, and the Medical Council were interviewed to gather data on factors affecting GP supply and demand. Purposive and snowball sampling methods were used to select participants with relevant managerial, executive, or research experience.

Six main categories of external factors were identified: social, political, technological, environmental, value-based, and economic. Social factors include demographic changes, social status of GPs, and job conditions. Political factors involve government policies, power dynamics, and political legitimacy. Technological factors encompass the impact of the internet, artificial intelligence, and medical equipment. Environmental factors relate to crisis preparedness and climate conditions. Economic factors include economic growth, inflation, and household income. Value-based factors cover cultural and family values and social perspectives on healthcare. Our framework explains how these external drivers interact across supply (entry, retention, geographic distribution) and demand (care-seeking, trust, affordability) pathways, offering concrete levers for policymakers to rebalance GP availability and utilization. Findings can guide targeted incentives, regulatory alignment, and technology-enabled primary care access to strengthen Iran’s GP workforce performance and population health outcomes.

**Supplementary Information:**

The online version contains supplementary material available at 10.1186/s12875-025-03123-2.

## Introduction

General practitioners, as the primary level of healthcare, play a key role in enhancing community health [1]. However, in recent years, the number and distribution of general practitioners in Iran have encountered challenges and changes [2]. Studies have shown that a significant portion of these challenges is attributable to factors outside the healthcare system—factors that are often connected to the country’s social, cultural, economic, and political conditions [3]. This issue can be analyzed from a supply and demand perspective in the field of general practitioners. Supply pertains to the number and recruitment or retention of general practitioners, while demand refers to the frequency of visits and the community’s need for these services [4]. Despite extensive debate on physician workforce planning, most evidence in Iran prioritises intra-system levers (training capacity, financing arrangements) and single-domain analyses. Less is known about how extra-system forces—macroeconomy, demographic change, sociocultural norms, political legitimacy, and technology diffusion—jointly drive GP labour market dynamics. This gap hampers coherent policy packages and system-level calibration of supply–demand balance.

From the supply side, social attitudes and values regarding the status of general practitioners are highly influential [5]. When a business-oriented or profit-focused perspective prevails in healthcare services, resulting in a decrease in the social capital of general practitioners, some of these doctors may lose their motivation to continue working in general practice [6]. Moreover, economic instability and inflation can cause medical students or recent graduates to favor higher-paying specialties over general medicine or to consider working abroad [7]. In this context, government policies regarding student intake capacity, the creation of financial incentives, and the facilitation of service delivery are also key factors in increasing or decreasing the supply of general practitioners [8].

Therefore, the aforementioned factors can directly or indirectly influence the entry of physicians into the general field and create conditions for decreasing or increasing supply. However, the dynamics of demand in the health system also depend on important economic, social, and technological variables [9]. Variables such as the purchasing power of the population, public awareness and trust in general practitioners, and transparency in the provision of medical services are effective. In situations where families are economically disadvantaged and healthcare costs impose a heavy burden on household budgets, visits to general practice clinics or private practices may decrease [10]. Additionally, when society’s attitude towards general practitioners is positive and they possess high professional credibility, people’s visits for primary and preventive treatments increase [11]. On the other hand, technological advancements such as electronic health records and appointment scheduling applications can improve access to these services by facilitating communication between citizens and general practitioners, thereby increasing demand [12]. In light of these considerations, this study investigates the following research question: Which external (extra-system) factors and mechanisms shape the supply (entry, retention, distribution) and demand (care-seeking, trust, affordability, access) for general practitioners in Iran? Our aim is to develop a systematic framework of these external factors and their interactions to inform workforce management and policy.

## Materials & methods

This study was conducted using a qualitative approach and by employing the conventional content analysis [13] method. Conventional content analysis is utilised when prior theory is limited and the researcher seeks to inductively derive categories from raw data; in our case, fragmented evidence on extra-system drivers of the GP labour market in Iran warranted an inductive approach to surface context-specific categories and mechanisms.

The study population consisted of key and informed individuals in the field of human resource management within Iran’s health system, who held direct or indirect responsibilities regarding the supply and demand of general practitioners at various levels (such as medical universities, the Ministry of Health, and the Medical Council). Additionally, a number of experts and specialists with research backgrounds or executive experience related to factors influencing the supply and demand of general practitioners were included in the study. Therefore, a purposive sampling [14] method was first employed; that is, individuals were selected based on their managerial and scientific backgrounds, ensuring they had more accurate information on the research topic. After conducting several preliminary interviews, snowball sampling [15] was also utilized to identify other qualified individuals and to expand data diversity; meaning that each interviewee introduced researchers to individuals who had sufficient qualifications and knowledge regarding the research themes. Inclusion criteria for the study included having relevant managerial or executive experience in the health system, awareness and understanding of the supply and demand of general practitioners, and willingness to participate voluntarily. Conversely, individuals who did not have sufficient willingness or opportunity to respond to questions were excluded from the research process.

In total, 27 individuals were selected in the first stage and interviewed. After that, when the initial review of the codes indicated that no new information was being generated, two additional interviews were conducted to further ensure and examine theoretical saturation. Ultimately, the analyses confirmed that the topic was fully saturated. Therefore, a total of 29 interviews were conducted as in-depth and semi-structured interviews to collect data(Table 1). Initially, the interview guide was developed based on previous literature and the research objectives. After the stage of familiarization and building trust with the participants, the interviews were primarily conducted in person at the workplace or a quiet location; in cases where in-person meetings were not possible, interviews were conducted by phone or online. The conversations were recorded with the verbal consent of the participants and then transcribed verbatim. To maintain confidentiality, the names and personal information of the individuals were coded. The average duration of each interview was approximately 50 min. Before starting each interview, the research objectives and the method of data storage were explained to the interviewee, and informed consent was obtained. All interviews were recorded with the consent of the interviewees and then transcribed verbatim.

**Table 1:** Demographic characteristics of interviewees

Variables	Number
Gender	
Male	23
Female	6
Age	
31–40	5
> 41	24
Education: General Practitioner	7
Education: Specialist Doctorate	22
Health services management, Public health, Health policy	12
Clinical specialty (e.g., Internal Medicine)	10
Work Experience	
11–20	11
> 21t	18

Conventional content analysis was employed for data analysis. The coding process began with an initial familiarization with the data, where the interviews were read multiple times carefully to allow the researchers to become acquainted with the text. In the initial coding phase, known as Open Coding, important statements and sentences were examined line by line, and key words or phrases were extracted. Each meaningful segment of the data was considered a “code.” In the stage of summarizing codes and forming subcategories, codes with similar themes were grouped and described under subcategories. Subsequently, in the stage of forming main categories, subcategories with thematic similarities were merged into several main categories through discussion and consensus among the research team. Our criteria for prioritizing or merging codes were their frequency and content significance in corroborating the research literature or understanding the phenomenon under study. In the final stage, the overall categorization of themes and categories was reviewed in several joint sessions with the research team, and any discrepancies were amended until consensus was reached. Finally, the codes and categories were organized using MAXQDA 2020 software, and an analytical report was developed.

To evaluate the quality and validity of the research findings, the criteria of credibility, transferability, dependability, and confirmability were considered. According to Lincoln and Guba [16], these criteria establish the trustworthiness of qualitative research: *credibility* refers to the truth value and confidence in the findings; *transferability* relates to the extent to which the results can be applied in other contexts; *dependability* concerns the consistency and stability of findings over time; and *confirmability* ensures that the results are derived from participants’ experiences rather than researcher bias [17]. To ensure credibility, sampling was conducted with maximum diversity to incorporate the various perspectives of different managers and experts. The extensive interaction of the researchers with the data collection and analysis process, the revision of interview transcripts, and the repeated listening to the recorded files helped deepen the understanding of the phenomenon. Member checking involved providing a selection of the extracted codes to some participants to verify the accuracy of the researcher’s interpretations. For transferability, a detailed description of the research context and the manner of conducting interviews (participants, location, and timing) was explained in the text so that readers could assess the extent to which the findings can be generalized or similar conditions apply in other contexts. In terms of dependability, an external audit was performed by sending copies of the codes and categories to two experts familiar with qualitative methods, and their feedback on the coding logic was reviewed and implemented in several joint sessions. The researchers (including healthcare management specialists and experts familiar with qualitative methods) collectively reviewed the codes and categories and discussed any disagreements until consensus was reached. To ensure confirmability, audio files, transcripts, and field notes were kept in the research archive to allow for re-auditing and tracking of the analysis process if needed. By adhering to the above criteria, the research documents and process were recorded in a documented and traceable manner, ensuring the confirmability of the result.

## Results

Based on the interview data, the supply and demand of general practitioners are influenced by external factors that lie beyond the direct control of health human resources management. We identified six main categories: social, political, technological, environmental, value-based, and economic factors (Table 2). These six categories represent inductively derived groupings that organised recurrent themes and mechanisms reported by participants and allowed us to map pathways to GP supply (entry, retention, distribution) and demand (care-seeking, trust, affordability, access).

**Table 2:** External factors of the health system affecting the supply and demand for physicians

Main Category	Subcategory	
Social Factors	Demographic and population factors	• Gender structure • Age structure and level of aging • Region and Geography • Population Distribution Status
	social structure and status	• The social status defined for a general physician • Community trust in general practitioners • The level of community acceptance regarding receiving services from general practitioners • The perspective of family and relatives towards the medical field • Ethnic issues • Advertising and social priorities (such as attention to beauty services)
	Social orientation	• Desire to study medicine • The level of societal specialization
	job conditions	• Personal and family living conditions • The impact of the medical profession on family.
Political factors	Policies	• International pressures • Government policies and programs • Security situation of the regions
	Power Dynamics	• Changes in policymaking due to government transitions • Political pressures
	Political legitimacy	• Space for free participation in social activities • The gap between people and policymakers • Social laws governing society
Technology Factors	Technology Factors	• Global Internet • Artificial Intelligence • Medical Equipment Technologies • • Internet of Things and Gadgets
Environmental factors	Environmental factors	• Forecasting for crisis periods such as disasters and epidemics • Weather conditions in different regions
Economic factors	Economic factors	• Economic growth (GDP) of the country • Economic instability (inflation) • Purchasing power of the people • Household income
Value Factors	Cultural and Value Factors (Values)	• Culture and values of family • Changes in the cultural and social fabric of society • Dominance of financial values • Prevailing culture and social perspectives in society • Public culture regarding the use of healthcare services

### Main category: social factors

Social factors were identified as one of the components in determining the supply and demand of general practitioners. In this context, interviews showed that demographic and population factors, social structure and status, Social orientation, and job conditions can have a significant impact on the supply and demand of general practitioners.

#### Subcategory: demographic and population factors

These factors refer to demographic characteristics such as age structure, gender distribution, geography, and population dispersion. According to the participants, the aging of society and the spatial distribution of the population can lead to changes in the level of demand.

“As the population ages, the demand for medical services increases.”(M4). This view clearly highlights the “demand” dimension, because an aging society requires more specialized and non-specialized care.

“ Spatial planning must be taken into account as a factor that determines how the population is distributed.”(M1). This statement indicates that the supply of physicians varies depending on population distribution; in areas with low population density, there is less incentive and fewer resources to establish a general practitioner, effectively reducing the supply. At the same time, potential demand may also remain unmet.

#### Subcategory: social structure and status

Interviewees often acknowledged that general practitioners, as the first point of patient contact, play an important role in fostering a culture of health and prevention. This social role not only increases public trust (on the demand side) but also motivates physicians as to whether they remain at this level or not (on the supply side).

“The value and status accorded to doctors in society has been very influential, allowing individuals to gain an identity and hold a social position.”(M1). This statement underscores the importance of the social status of general practitioners for both patients (demand) and the physicians themselves (supply). If communities place a high value on general practitioners, people will trust them before consulting specialists, and in turn, physicians will be inclined to remain in that role.

“ Everyone says that people trust general practitioners, but in big cities, people go straight to specialists or subspecialists, as if they don’t consider general practitioners sufficient.”(M7). This quote reveals a contradictory aspect: in metropolises with greater access to specialists, demand for general practitioners decreases.

In various parts of the country, ethnic-related issues can negatively affect the presence of general practitioners. One of the interviewees stated that “employing women in tribal, ethnic, and border areas is not advisable, because the doctor “would not even dare to leave the residence to go out and visit patients.”(M8). Insecure conditions in ethnic and tribal areas, especially for female doctors, impact both the reduction in supply, meaning the decreased motivation and ability to provide services, and the diversion of demand through reduced effective visits or reliance on alternative methods.

“These days, many doctors have started working with lasers and are doing very good advertising… Advertising has a strong influence on people’s values.” (M11). From the time this issue is raised, it diminishes general practitioners’ motivation to stay involved in preventive and primary care, because advertising in the beauty field demonstrates higher earnings, and over time, people become more inclined toward cosmetic services, which diverts demand away from general practitioners.

#### Subcategory: social orientation

The social appeal of the field of medicine initially attracts many individuals to general medicine (increasing short-term supply). However, due to the specialist-oriented structure of the healthcare system and graduates’ personal inclinations toward specialization, many of these general practitioners ultimately pursue specialties in certain fields, leaving some specialty areas with vacancies. As a result, the supply of general practitioners to meet the demand for primary healthcare services at the community level decreases. This contradiction between the “real need for general practitioners” and the “minimal economic and credit attention to general medicine compared to specialization” represents the point of tension between the supply and demand for general practitioners.

According to the interviewee’s statement, “students tend to pursue studies in medicine after the university entrance exam, and this is evident in the competition to take the exam and in the selection of the medical field.“(M3). It is clear that the field of medicine holds significant appeal and value in the public consciousness. This initial inclination leads to an increase in the number of entrants into the medical field (and consequently a potential rise in the supply of general practitioners in the short term).

“Despite the increase in the number of general medicine graduates, the health system is ‘specialist-oriented’ and places a high value and rewards (financial, prestige, and social status) on specialization.“(M9).

#### Subcategory: job conditions

Job conditions include the alignment between what medical professionals learn during their education and the actual needs of society, the location of service, and the impact on personal and family life, as well as issues such as job difficulty, which directly affects the willingness of general practitioners to work in this field (supply side). Additionally, the quality of the services provided is also related to these conditions, which may change the community’s perception of this profession (demand side).

“There is a disconnect between what individuals learn during their medical education and the needs of society after graduation.”(M14). This perspective cites that when general practitioners encounter straightforward outpatient illnesses in real-life settings, they may feel that their training, largely centered around complex diseases in hospitals, does not apply effectively. As a result, some of these doctors may choose to transition into other fields.(Decrease in supply).

“In the distribution of legal commitments in the medical field, no attention is paid to the individual’s place of residence. The person’s home is located elsewhere, and they are sent to work in different places… What happens to this individual’s living conditions and the negative impact on their family?“(M10). A significant part of the “shortage of supply” of general practitioners in remote or disadvantaged areas is due to this compulsory or unintended transfer. Such a difference also affects demand; people in the region may not have local doctors available and may effectively be deprived of services.

“Doctors are away from their families for long hours due to extended shifts and spending long hours in their practices, and this has affected childbearing, child upbringing, and family stability.”(M11). Based on this, graduates of the medical field may abandon general practice and move toward less stressful professions. Consequently, the supply of general practitioners may decrease in the long term.

### Main category: political factors

Political factors are recognized as one of the main categories influencing the supply and demand of general practitioners. These factors directly and indirectly affect the distribution of human resources within the healthcare system. Therefore, understanding and analyzing these factors and their subcategories, especially in the context of the supply and demand for general practitioners, is of great importance. Policymakers and decision-makers must design and implement effective measures to improve the healthcare system, taking these factors into account. In this regard, elements such as policies, power dynamics, and political legitimacy have been identified as subcategories of this category.

#### Subcategory : policies

Policies, as the first subcategory, are communications, programs, and governmental strategies that can lead to the establishment of medical service needs and supply. Changes in health and treatment policies can affect the number of general practitioners and their distribution patterns in different regions. Based on an analysis of the interviews conducted, three codes were identified in this study: international pressures, government policies and programs, and the security situation in the regions.

One of the important factors affecting the supply of general practitioners is international pressures. These international pressures can lead to an increase in the migration of general practitioners to countries with better job opportunities. One of the interviewees mentioned, “International pressures are felt significantly compared to countries like the United States and Oman. Many specialists decide to migrate due to the reduced international pressures resulting from the dollar. These individuals, given the existing pressures in Iran, can convert their income to dollars and send it to their families.“(M18).

Based on the interviews conducted, experts stated that the government’s policies and programs are one of the key factors influencing the supply and demand for general practitioners. These policies directly affect the number of general practitioners and their geographical distribution. One of the participants noted that “government policies regarding health and healthcare, particularly in determining the number of medical student admissions and the working conditions for general practitioners, significantly impact the supply and demand for this occupational group. If the government does not provide supportive programs and incentives for general practitioners in underserved areas, we will witness a decrease in physicians’ willingness to work in these regions.“(M14).

The security situation in the region is one of the important factors that directly affects the supply and demand for general practitioners. In areas facing armed conflicts, social unrest, and security issues, general practitioners encounter specific challenges that can influence their decisions to work in these regions. One interviewee clearly stated that “in provinces like Khuzestan and Kerman, the security situation—particularly in tribal areas and armed conflicts—has a significant impact on the supply of general practitioners. Due to security risks and unrest in the Sistan and Baluchestan region, general practitioners tend to be reluctant to work in these areas, while the demand for medical services significantly increases in unsafe regions because of incidents and conflicts.“(M10).

#### Subcategory: power dynamics

Power dynamics refer to a set of institutions and policymakers that influence the supply and demand for general practitioners. Political changes and health programs that arise from shifts in government can affect the needs of society, thereby altering the demand for medical services. Generally, policies and the influence of power institutions can impact both the supply and demand for general practitioners. One interviewee stated that “new individuals who come into power pursue different approaches and programs that significantly affect the participation of actors in this field; for instance, in the referral system and family physician programs, the role of general practitioners has become more prominent. With the implementation of the transformation plan, the presence of specialists has also increased. In our country, all choices and legal decisions related to power dynamics are influenced by political factors.“(M17).

#### Subcategory: political legitimacy

Political legitimacy, as a key factor in shaping the supply and demand for general practitioners in Iran, has a significant impact on public trust in the healthcare system. When the political system appears valid and legitimate, people are more likely to trust general practitioners and health advisors, and this trust can lead to an increase in demand for medical services. Analyzing the interviews revealed three codes: the space for free participation in social activities, the gap between the people and policymakers, and the Social laws governing society.

General practitioners can play an active role in the political and social structures of the country, especially in institutions such as city and village councils and parliament. This participation can lead to an increase in demand for medical services, as when general practitioners are involved in decision-making and social processes, the trust that people have in them and the healthcare system grows. This trust encourages people to seek out the services of general practitioners more and to rely on them for advice and treatment. However, one participant stated, “The question is how much opportunity is given to general practitioners to participate in these social processes? Unfortunately, the activities of general practitioners are largely limited to professional tasks.“(M15).

The gap between the people and policymakers has a significant impact on the supply and demand for general practitioners’ services. This gap can negatively affect the demand for general physicians’ services. When people do not trust the policies and programs of the healthcare system related to general medicine, they tend to visit general practitioners less frequently. One interviewee stated: “The deep divide between the public and policymakers, especially concerning the new generation, leads to a lack of trust and acceptance of general practitioner policies, including family medicine.“(M8). As a result, this factor not only reduces the supply of services but also has a negative impact on the demand for medical services, as a lack of trust in the healthcare system can lead to a decrease in people’s visits to general practitioners.

Social laws governing society play a significant role in the supply and demand for public medical services. Social laws should be designed in a way that supports suitable and valuable professions for promoting community health. If society gives more importance and value to health-related professions, especially public medicine, and views violations of these laws as undesirable, this can lead to an increase in demand for medical services. In such an environment, people will be more likely to consult general practitioners and place greater trust in their services. On the other hand, if the prevailing laws in society allow for socially undervalued professions—such as cosmetic medicine—to dominate, this can negatively impact the supply and demand for public medicine. One interviewee pointed out the importance of this issue: “To create a flourishing community, social laws must be such that violations are not regarded as a value.“(M13).

### Main category: technology factors

Technology is increasingly changing the needs and expectations of people, and these changes have particularly affected the supply and demand for general practitioners. With the emergence of new treatment methods and information and communication technologies, the type of services provided by general practitioners is moving towards improved quality and increased access.

#### Subcategory: technology factors

The global Internet, as an effective technological factor, has profound impacts on the supply and demand for general practitioners. One participant mentioned: “The global Internet has increased access to medical information. These changes not only raise public awareness but can also lead to improved quality of medical services and increased demand for general practitioners.“(M5). Patients, due to the abundance of information available on the internet, are turning more than ever to medical consultations and services. This access to information increases the demand for general practitioners.

Artificial intelligence is another factor identified in the technology sector that influences the demand for general practitioners, as it utilizes algorithms and software to analyze complex medical data. One participant stated, “In the near future, artificial intelligence will replace general practitioners.“(M10). Concerns about the potential replacement of physicians by artificial intelligence may lead to a decreased willingness among patients to seek in-person consultations and an increased inclination towards AI-based healthcare services, which could result in a reduced demand for general practitioners.

Medical equipment technologies have a broad impact on the demand for general practitioners. One participant stated, “Medical equipment technologies increase diagnostic accuracy and the quality of treatment.“(M12). This could significantly increase patients’ trust in general practitioners. Patients are looking for effective and reliable treatments, and this trust can lead to a greater willingness to seek out general practitioners and a higher likelihood of returning to them. One of the main reasons patients consult specialists instead of general practitioners is related to diagnosis. However, if general practitioners can effectively diagnose diseases, this could reduce the burden on specialists. As a result, all these factors could lead to increased demand for general practitioners and a greater need for more of them in the community.

“The Internet of Things and medical gadgets allow us to continuously monitor patients’ health status and establish more effective communication with them”.(M4). These factors can directly increase the demand for general practitioners. As patients become more aware of their health status through continuous monitoring, they are encouraged to visit general practitioners more frequently.

### Main category: environmental factors

#### Subcategory: environmental factors

Environmental factors refer to the set of characteristics and conditions that influence activities and decision-making related to the medical field. Examining and understanding environmental factors helps us better identify the health needs of the community and provide better planning for the delivery of medical services. Therefore, paying attention to these factors is essential in public health policymaking and the training of physicians.

One of the most important environmental factors is preparedness for times of crisis, such as disasters and epidemics. One of the interviewees stated, “One of the factors we need to consider for assessment is, if a crisis occurs, like the coronavirus crisis that affected the entire country, how many general practitioners and infectious disease specialists we should have available during this crisis.” This statement indicates that insufficient planning during crises can have serious consequences for the health system.(M4).

The climatic conditions of different regions are also among the environmental factors that influence the supply and demand for doctors. One of the interviewees stated that “climate change can lead to an increase in the number of vulnerable individuals who require medical care. This population increase will create additional pressure on existing medical services.“(M9).

### Main category: economic factors

An economic factor refers to a set of financial and economic conditions and variables that influence the behavior and decision-making of individuals and institutions in the field of healthcare. This factor is recognized as one of the main determinants of the supply and demand for general practitioners and encompasses various aspects that directly or indirectly affect access, quality, and types of medical services. Based on conducted interviews, indicators such as economic growth rates (GDP) of the country, economic instability (inflation), purchasing power of the people, and household income were identified.

#### Subcategory: economic factors

Economic growth and Gross Domestic Product (GDP) are among the significant factors affecting the supply and demand for general practitioners. One of the participants stated that “the country’s economic situation has a substantial impact on the number and accessibility of general practitioners. Currently, with the decline in economic growth, which is a quantitative variable, and the increase in the government’s budget deficit, we are observing its negative effects on the supply and demand for doctors in various ways.“(M11). The lack of sufficient financial resources for hiring and retaining doctors leads to a decreased interest in entering this profession and the emigration of doctors to other countries. On the other hand, in conditions of poverty and economic inequality, the need for medical services may increase, as chronic diseases caused by poverty become more prevalent. However, financial constraints and patients’ inability to afford treatment costs can result in a reduced number of visits to general practitioners.

Economic instability and inflation have different impacts on the supply and demand for general practitioners. One of the interviewees stated that “in times of economic instability and inflation, many individuals avoid visiting healthcare centers due to costs.“(M6). This change in behavior leads to an imbalance in the demand for various medical services.

The purchasing power of the public plays a significant role in determining the demand and supply for general practitioners. One participant stated that “the rise in inflation and the decrease in purchasing power lead to a reduction in demand for health services and visits to healthcare centers. This decrease in visits also directly affects the demand for labor in the health sector.“(M2) The decline in demand for healthcare services not only affects the number of patients but also impacts the supply of general practitioners due to the lack of job appeal and financial opportunities.

Household income is one of the factors affecting the supply and demand for general practitioners. One participant stated that “in households with higher incomes, individuals generally have better access to healthcare services and show a greater willingness to consult general practitioners.“(M11). Households with lower incomes avoid visiting doctors due to financial concerns and only turn to medical services in emergencies. This decrease in demand for medical services not only weakens the job market for general practitioners but also leads to a loss of motivation and retention among the existing doctors in the system.

### Main category: value factors

Value factors are referred to as criteria that affect individuals’ perceptions and evaluations of the supply and demand for general practitioners. Based on the analysis of conducted interviews, a subcategory of value and cultural factors (values) was identified.

#### Subcategory: cultural and value factors (values)

In this category, five codes were identified, including the Culture and values of family, Changes in the cultural and social fabric of society, Dominance of financial values, Prevailing culture and social perspectives in society, Public culture regarding the use of healthcare services.

One of the factors influencing the supply and demand for general practitioners is family culture and values. Family culture can lead to an increase in the number of general practitioners, as in societies where families place a high value on the medical profession, young people are more inclined to enter this field. On the other hand, family culture also affects the frequency of visits to general practitioners. One of the interviewees stated that “family culture and values play a significant role in the career choices of general practitioners. In societies that respect physicians, young people are more inclined to pursue a career in medicine; however, family pressures and expectations can negatively impact their persistence in this profession. Thus, family culture directly and indirectly influences the supply and retention of general practitioners.“(M4).

Social changes have led families to modernize their health approaches and seek access to general practitioners for preventive and therapeutic consultations. On the other hand, cultural changes have increased the need for general practitioners for nutritional and public health advice. Social and cultural context changes are driving demand. One participant stated, “Cultural and social transformations have various impacts on communities, but in our country, one of the effects of these changes is the demand for doctors due to the influence of Western culture on lifestyle and diet.“(M7).

The dominance of financial values is another factor that affects the supply and demand for general practitioners. When general practitioners prioritize earning a high income, they tend to focus more on providing high-cost services, which can impact the demand for medical services. One interviewee stated, “In a situation where financial values prevail in society, many doctors view medical practice solely as a source of income, leading to a decline in the previous sanctity of this profession in serving people and treating patients. This change in perspective causes some doctors to create induced demand to increase their income.”(M11).

Unfortunately, there is a common perception that general practitioners have less value and credibility compared to specialists. This attitude not only harms the morale of general practitioners but also affects the quality of healthcare services. If we want to have an effective healthcare system, we must pay attention to the equal value and credibility of all physicians, whether general or specialist.(M8).

The culture of the people influences individuals’ attitudes toward healthcare services and physicians. In regions where visiting a doctor is considered a harmful issue for cultural and social reasons, patients may avoid seeking medical services, leading to a decrease in demand for general practitioners. Additionally, a tendency to focus on treatment rather than prevention can result in less demand for regular check-ups and preventive consultations, which negatively impacts public health and accessibility to timely treatment. One of the participants stated, “People view healthcare services through the lens of their culture. In some areas, patients avoid visiting doctors because they consider it harmful for cultural reasons. The culture of the people leans more towards treatment than prevention, which affects demand for doctors.“(M6).

## Discussion

Our findings indicate that multiple factors exist outside the direct control of the health system, which influence the supply and demand of general practitioners. These factors can be broadly categorized into six main groups: social, political, technological, environmental, value-based, and economic factors.

Demographic factors play an important role in determining the supply and demand for general practitioners. Based on the findings of the present study, gender structure, age structure, and the distribution of the population have an impact on the supply and demand for general practitioners, which was confirmed by the study conducted by Graaf-Ruizendaal [18]. Changes in population structure, such as the increasing age of the population and the dispersion of the population, impact the demand for medical services. As the population ages, the need for medical services rises. A study aligned with the present one indicated that the age structure of the population is a key factor in shaping the future landscape of healthcare and the demand for general practitioners. With demographic shifts toward an older population, healthcare systems must adapt to ensure an adequate supply of physicians, mitigate potential shortages, and provide more comprehensive care to the aging patient population [19]. One of the social factors emphasized in this study is the impact of trust and acceptance of general practitioners on the demand for services from them, which has significant implications for estimating the need for general practitioners. Consistent with the results of this study, other research has also shown that when patients trust their doctors, they are more likely to seek medical services [20, 21]. If patients feel that their general practitioner is trustworthy, the likelihood of them returning to that doctor increases, which in turn significantly boosts the regard for general practitioners in the community. Another study showed that in countries with strong referral systems and high levels of trust in general practitioners, the demand for general medical services is usually higher [22]. This is mainly because citizens are more inclined to consult general practitioners, as they know that if necessary, they will receive appropriate referrals to specialists, which increases access to and overall satisfaction with healthcare services.

Political factors, including policies, power dynamics, and political legitimacy, directly and indirectly shape the distribution of human resources in the healthcare system and influence the availability and access to general practitioner services. A study conducted by Raeve PD, in alignment with this present study, examined the role and impact of government policies on the supply of human resources in the health sector. The results indicated that the provision of human resources is affected by political factors. Political decisions influence the number, distribution, and quality of the healthcare workforce, particularly in areas such as education, licensing, and the hiring of physicians [23].

According to the findings of the present study, changes in political priorities and exigencies impact the allocation of resources and the supply and demand for general practitioners. Accordingly, results from a study in Britain showed that policymakers should adopt more integrated approaches to the workforce and strive to improve the employment and educational conditions of staff while addressing changing demands [24]0.0 Emphasizing the recruitment and retention of general practitioners, especially in key areas such as primary care, should be prioritized to improve public health.

Based on the findings of the present study, another factor influencing the supply and demand for general practitioners is technology. Technological advancements such as electronic health records, artificial intelligence, and the Internet of Things significantly impact the healthcare system, including the needs and expectations of patients and the demand for services. These changes have a direct effect on the number and types of services offered by general practitioners. Results from various studies in this area indicate that technologies like electronic health records [25, 26] and artificial intelligence enable general practitioners to improve the quality of care and increase patient visits [27–29]. Additionally, digital health technologies, including telemedicine, have the potential to enhance access to primary care services that can be provided by general practitioners. Therefore, innovations in digital health will directly affect the supply and demand for general practitioners in the future, and policymakers and leaders must ensure that general practitioners can effectively utilize and benefit from these technological advancements.

Environmental factors play a significant role in the healthcare system as one of the influential components affecting the supply and demand for general practitioners. Among these factors are the preparations for times of crises, such as disasters and epidemics that occur suddenly. These factors influence the planning for human resources and, in general, the supply and demand for doctors [30]. Penelope Burns also emphasizes, in line with the current study, the role of general practitioners in strengthening health and healthcare systems during crises. A better definition of the role of general practitioners in disaster response, along with greater support and integration within these systems, not only aids in improving disaster response but also enhances the quality of healthcare services in critical conditions [31]. Crisis and disaster conditions have a significant impact on supply and demand within the profession of general practitioners. These conditions necessitate planned preparation and flexibility to ensure human resources. Another environmental component that affects the supply and demand for physicians is extreme climate changes and the occurrence of natural disasters. In this regard, Grant Blashki’s research demonstrated that climate change, as a global public health issue, has extensive effects on the supply and demand for medical services [32]. On one hand, climate change leads to an increase in heat-related illnesses, infectious diseases caused by environmental changes, and mental health issues, which raises the demand for medical services. On the other hand, experienced general practitioners with their unique way of thinking can play an important role in managing this crisis.

One of the factors impacting the supply and demand for general practitioners is economic factors, which are recognized as one of the main pillars of healthcare systems in countries. The economic growth (GDP) of a country, the instability of the economic situation, especially during inflation, the purchasing power of the people, and household income have significant effects on decision-making related to the provision and delivery of medical services. Various studies, such as a study by Scheffler RM (2008), indicate that an increase in the inflation rate and a decrease in the purchasing power of the people can lead to a reduction in the demand for medical services [33]. Sustainable economic growth, in turn, leads to increased demand and consequently an increase in the supply of general practitioners. Similarly, compared to other reputable sources such as (Dey S, 2018), it can be seen that countries that have managed to achieve sustainable economic growth while also paying attention to the improvement of household living conditions have witnessed an increase in access to physicians and quality medical services [34].

One of the key factors that influences the supply and demand for general practitioners is the value factor, which can aid in a better understanding of the dynamics of the healthcare service market. The cultural values of society, which affect the choice of profession and the type of services provided by physicians, are particularly significant in many communities, especially in developing countries. These factors, emphasizing individual tendencies such as the desire to serve the community and receive positive impacts from professional activities, can increase motivation and, consequently, the supply of general practitioners. A study conducted in this area confirms that the relationship between values and the professional behaviors of physicians can play a determining role in shaping the supply and demand for this group [35]. In societies where the values of cooperation and community service are more prevalent among physicians, not only is the quality of healthcare services better, but there is also an increased inclination to engage in the field of general medicine [36]. Conversely, a deficiency in these values can lead to a reduced supply and ultimately dissatisfaction in the market for general practitioners.

While much of the literature aligns with our findings regarding demographic ageing, trust in GPs, and the salience of political and economic levers [13–19, 28–31], several studies have reported differing emphases or partially divergent patterns that nuance the external-driver view presented here: In some metropolitan contexts with abundant specialist access, studies have observed sustained direct specialist consultations despite primary care gatekeeping messages, suggesting weaker elasticity of demand toward GPs than expected under referral norms. This contrasts with settings where trust and referral protocols more reliably channel first-contact care to GPs. Possible explanations include entrenched specialist-centric cultures, perceived diagnostic advantages, and insurance design favouring direct specialist entry.

While many analyses indicate digital tools, EHRs, and telehealth can augment GP reach and visits [20–24], other reports highlight substitution risks when AI-enabled triage or consumer digital pathways bypass GPs for niche services. Contextual moderators—payment models, scope-of-practice rules, and patient digital literacy—appear to determine whether technology complements or displaces GP roles.

### Study limitations and strength

The study on external factors influencing the supply and demand for general practitioners (GPs) in Iran presents notable strengths and limitations. Strengths include a comprehensive framework that categorizes six main external factors (social, political, technological, environmental, value-based, and economic), which effectively encapsulates the complexities impacting GPs within the healthcare system. The qualitative approach allows for rich, in-depth insights through interviews with 29 experts, enhancing the validity of the findings. Additionally, the rigorous methods used for ensuring credibility, such as member checking and external audits, bolster the reliability of the research. However, the study also has limitations; its context-specific findings may not be generalizable to other regions or healthcare systems due to differing social and political landscapes. Furthermore, potential biases in participant responses, given their close ties to the healthcare system, could affect the objectivity of the data. Lastly, focusing solely on qualitative data may overlook valuable quantitative insights that could further enhance understanding of the supply-demand dynamics in healthcare.

## Conclusion

The present research examines the external factors influencing the supply and demand for general practitioners in Iran. The findings indicate various complexities that affect the supply and demand situation for general practitioners. To improve the current status, it is essential that policymakers and health system managers pay attention to all these factors and design appropriate policies that can enhance the conditions for general practitioners and facilitate achieving the community’s health objectives. Attention to social factors, as one of the main components determining the supply and demand for general practitioners, particularly demographic and population factors, the structure and social status of general practitioners, and social trends directly impact the need for medical services. An aging population and an increase in chronic diseases naturally raise the demand for medical services. Additionally, the community’s trust in general practitioners and their acceptance as health advisors significantly influences patients’ visits to this group of doctors.

Government health and medical policies, international pressures, and the security situation in regions directly influence the distribution of human resources in the health system. Especially in underserved areas, supportive policies and appropriate incentives can help increase doctors’ willingness to work in these regions. Therefore, attention to these factors is essential in designing and implementing health policies.

Technological advancements, particularly in the fields of information and communications, have profound impacts on the supply and demand for general practitioners. Technologies such as artificial intelligence, the Internet of Things, and advanced medical equipment not only enhance the quality of medical services but also increase the ability of general practitioners to provide services to patients.

Environmental factors have also been identified as one of the important components in the supply and demand for general practitioners. Forecasting for periods of crises and climate conditions in different areas can improve planning for human resource provision in health care. During crises, such as disease outbreaks, the demand for general practitioners significantly increases, necessitating precise planning and the provision of suitable human resources.

Economic factors are recognized as one of the main pillars influencing the supply and demand of general practitioners. Economic growth, instability in the economic situation, and people’s purchasing power have significant impacts on access to medical services.

Changes in values and social attitudes, especially among the younger generation, profoundly affect the decision to choose a medical career and the willingness to serve in this profession. Furthermore, the commercialization of the medical profession and the decline in physicians’ social capital can negatively impact the quality of services provided. Therefore, there is a need for cultural promotion and enhancement of the social values associated with the medical profession and its importance in society.

Based on the findings of this study, it is recommended that supportive policies and appropriate incentives for general practitioners be established, especially in underserved areas. Attention to improving economic conditions and increasing people’s purchasing power could help increase demand for medical services and improve access to healthcare. The need for cultural promotion and enhancement of social values related to the medical profession and its significance in society is felt. This can contribute to improving the public image of physicians and increasing the community’s trust in them.

## Supplementary Information

Supplementary Material 1

## Data Availability

All data generated or analyzed during this study are available from the corresponding author upon reasonable request via email.

## References

[1] Selman S, Siddique LJOJSS. Role of primary care in health systems strengthening achievements, challenges, and suggestions. 2022;10(10):359–67.

[2] Doshmangir L, Shirjang A, Assan A, Gordeev VSJPHCR. Development. Iranian primary health care network: challenges and ways forward. 2023;24:e1. 10.1017/S1463423622000354 PMC9884526 36617866

[3] Summit GA, Knebel EJHPEABQ. Challenges facing the health system and implications for educational reform. 2003;192.

[4] Parisi R, Lau Y-S, Bower P, Checkland K, Rubery J, Sutton M, et al. GP working time and supply, and patient demand in England in 2015–2022: a retrospective study. Br J Gen Pract. 2024. 10.3399/BJGP.2024.0075. 39284685 10.3399/BJGP.2024.0075 PMC11423349

[5] Robinson G, Gould MJBJGP. What are the attitudes of general practitioners towards research? 2000;50(454):390–2. PMC1313705 10897538

[6] Crowley R, Atiq O, Hilden D, Health P, PPCotACo, Atiq O et al. Financial profit in medicine: a position paper from the American college of physicians. 2021;174(10):1447–9. 10.7326/M21-1178 34487452

[7] Phillips JP, Wilbanks DM, Rodriguez-Salinas DF, Doberneck DMJME. Specialty income and career decision making: a qualitative study of medical student perceptions. 2019;53(6):593–604. 10.1111/medu.13820 30821014

[8] Simoens S, Hurst JJOP. The Supply of Physician Services in OECD Countries. OECD Health Working Papers, No. 21. 2006.

[9] Ying S, Leone D, Cicchiello AF, Cicchiello AF, Kazemikhasragh A. J. JJoB. Industrial dynamics and economic growth in health-care context. Evidence from selected OECD countries. J Bus Ind Mark. 2022;37(8):1706–16.

[10] Hajibadali P, Nadrian H, Hashemiparast MJJoHE Promotion H. Challenges of implementing the family physician program: a qualitative study in an Iranian urban community. J Hum Environ Health Promot. 2023;9(4):216–23.

[11] Huang EC-H, Pu C, Chou Y-J, Huang NJITJHCO. Provision, Financing. Public trust in physicians—health care commodification as a possible deteriorating factor: cross-sectional analysis of 23 countries. 2018;55:0046958018759174. 10.1177/0046958018759174 PMC5843089 29502479

[12] Piot PJGph. Innovation and technology for global public health. 2012;7(sup1):S46-S53. 10.1080/17441692.2012.698294 22780442

[13] Hsieh H-F, SEJQhr S. Three approaches to qualitative content analysis. 2005;15(9):1277–88. 10.1177/1049732305276687 16204405

[14] Campbell S, Greenwood M, Prior S, Shearer T, Walkem K, Young S, et al. Purposive sampling: complex or simple? Res Case Examples. 2020;25(8):652–61. 10.1177/1744987120927206 PMC7932468 34394687

[15] Ting H, Memon MA, Thurasamy R, Cheah J-HJAJoBR. Snowball sampling: a review and guidelines for survey research. Asian J Bus Res. 2025. 10.14707/ajbr.250186.

[16] Lincoln YS, Guba EG. Naturalistic inquiry: sage; 1985.

[17] Shenton AKJEfi. Strategies for ensuring trustworthiness in qualitative research projects. 2004;22(2):63–75.

[18] de Graaf-Ruizendaal WA, de Bakker, DHJHrfh. The construction of a decision tool to analyse local demand and local supply for GP care using a synthetic Estimation model. 2013;11:1–9. 10.1186/1478-4491-11-55 PMC4231547 24161015

[19] Markit IJAoAMC. The complexities of physician supply and demand: Projections from 2015 to 2030. 2017.

[20] Khorasani E, Keyvanara M, Karimi S, Jazi MJJI. Views Health Syst Experts Macro Factors Induc Demand. 2014;5(10):1286. PMC4223949 25400888

[21] Verhaak PF, Brink-Muinen Avd, Bensing JM, Gask LJTEJPH. Demand and supply for psychological help in general practice in different European countries: access to primary mental health care in six European countries. 2004;14(2):134–40. 10.1093/eurpub/14.2.134 15230497

[22] Li L, Kuang LJCGP. Framework of the referral system in primary care practice. 2018;21(4):375–81.

[23] Raeve Pd, Xyrichis A, Recio S, Clocchiatti A. The politics of health workforce planning and forecasting. 2014.

[24] Anderson M, O’Neill C, Clark JM, Street A, Woods M, Johnston-Webber C et al. Securing a sustainable and fit-for-purpose UK health and care workforce. 2021;397(10288):1992–2011. 10.1016/S0140-6736(21)00231-2 PMC9634455 33965066

[25] Nikitenko V, Voronkova V, Kozar Y, Oleksenko R, Yanchevskyi O, Korobko IJRZ. Digital healthcare in the context of challenges and opportunities of technological progress in the countries of the European union. 2023;14(40):315–33.

[26] Pagliari C. J. Jogh. Digital health and primary care: past, pandemic and prospects. J Glob Health. 2021;11:01005. 34221352 10.7189/jogh.11.01005 PMC8251683

[27] Mills J, Fox J, Damarell R, Tieman J, Yates, PJBpc. Palliative care providers’ use of digital health and perspectives on technological innovation: a National study. 2021;20(1):124. 10.1186/s12904-021-00822-2 PMC8349145 34364379

[28] Borges do Nascimento IJ, Abdulazeem H, Vasanthan LT, Martinez EZ, Zucoloto ML, Østengaard L et al. Barriers and facilitators to utilizing digital health technologies by healthcare professionals. 2023;6(1):161. 10.1038/s41746-023-00899-4 PMC10507089 37723240

[29] Pote H, Rees A, Holloway-Biddle C, Griffith EJDH. Workforce challenges in digital health implementation: how are clinical psychology training programmes developing digital competences? Digit Health. 2021;7:2055207620985396. 33628457 10.1177/2055207620985396 PMC7883155

[30] Burns PL, FitzGerald GJ, Hu WC, Aitken P, Douglas KAJP, Medicine D. General practitioners’ roles in disaster health management: perspectives of disaster managers. 2022;37(1):124–31. 10.1017/S1049023X21001230 34857062

[31] Burns P, Douglas K, Hu W, Aitken P, Raphael BJAJGP. General practitioners in the field:‘A qualitative study of general practitioners’ experiences in disaster healthcare’. 2020;49(3):132-9. 10.31128/AJGP-08-19-5054 32113212

[32] Blashki G, Abelsohn A, Woollard R, Arya N, Parkes MW, Kendal P et al. General practitioners’ responses to global climate change-lessons from clinical experience and the clinical method. 2012;11:1–5. 10.1186/1447-056X-11-6 PMC3469365 22873633

[33] Scheffler RM, Liu JX, Kinfu Y, Dal Poz MR. Forecasting the global shortage of physicians: an economic-and needs-based approach. JBotWHO. 2008;86(7):516-23B. 10.2471/BLT.07.046474 PMC2647492 18670663

[34] Dey SJ. The nexus between population growth rates, number of registered doctors, number of health complexes and economic growth in Bangladesh: a VECM analysis. TMUJ. 2018;3(1):22–32.

[35] Clark PG. Values in health care professional socialization: implications for geriatric education in interdisciplinary teamwork. Gerontologist. 1997;37(4):441–51. 9279032 10.1093/geront/37.4.441

[36] Stevens RAJTMQ. Public roles for the medical profession in the united states: beyond theories of. Decline Fall. 2001;79(3):327–53. 10.1111/1468-0009.00211 PMC2751200 11565160

