# Supplementary material for: The role of external factors in shaping the supply and demand of general practitioners in Iran: a qualitative study
Source: BMC Prim Care. 2025 Nov 26;27:2. doi: 10.1186/s12875-025-03123-2 (PMC12764044; doi:10.1186/s12875-025-03123-2)
Supplement: Supplementary file 1 — Supplementary Material 1 [file 12875_2025_3123_MOESM1_ESM.docx]

In the Name of God

Estimating the number of general practitioners required for the health system of Iran with a system dynamics approach

Introduction The health sector, considering the extensive range of services and the importance of its goals to achieve health for all and social justice, requires the appropriate training and distribution of human resources across all spatial and temporal dimensions needed by the community. Human resources, specifically physicians, are one of the most important assets of the healthcare delivery system. Estimating the number of healthcare professionals is particularly significant due to the sensitivity of the medical graduates' field, which is directly related to the health of the community.

Training the right number of physicians requires an accurate estimation of the country’s needs. Estimating the need for physicians in different regions of the country enables proper distribution of the workforce. Globally, various methods and models have been designed and implemented to estimate healthcare human resources. Each of these models is tailored to specific temporal and spatial conditions; therefore, their application in communities with differing conditions is not suitable. The ever-increasing changes in social, political, cultural, economic, knowledge and technology conditions, and disease patterns lead to shifts in the needs and demands of the community for services, as well as the methods and tools for delivering those services. This changing landscape necessitates the application of suitable estimation methods and models.

Based on the reviewed studies, no research has been conducted in Iran on estimating the general practitioner workforce with a system dynamics approach. The aim of this study is to estimate the required number of general practitioners for the Iranian health system using a system dynamics approach. This study will be conducted based on a system dynamics framework and will employ group modeling in five phases. Initially, the dynamic phenomenon will be defined, followed by a precise definition of the issue and the identification of influential variables, resulting in the creation of a conceptual model and dynamic hypothesis. After formulating the hypothesis, cause-and-effect diagrams, as well as states and flows, will be drawn and modeled with mathematical equations, ultimately leading to the development and examination of probable and desirable scenarios. In this study, through document analysis, in-depth interviews, literature review, and group modeling, the dynamic hypothesis of the system will be defined. The findings of this research will provide a roadmap for decision-making and planning regarding human resources, aiming to change and improve the performance of the health system and will represent an important step toward offering a draft to the Ministry of Health of Iran.

Questions

1. Part One Questions: For the external factors of the health system: (SPTEEP.V) 1.1. What are the social factors influencing the supply and demand for general practitioners? (Social status, education system, value system, gender) 1.2. What are the political factors affecting the supply and demand for general practitioners? (Government policies on training and managing general practitioners, Cultural Revolution Council’s influence on increasing or decreasing admissions, preventing the importation of Pakistani physicians) 1.3. What are the technological factors affecting the supply and demand for general practitioners? 1.4. What are the environmental factors affecting the supply and demand for general practitioners? 1.5. What are the economic factors impacting the supply and demand for general practitioners? (Future job income, attracting neighboring countries with high income) 1.6. What are the value-based factors affecting the supply and demand for general practitioners? (Gender, ethical considerations, beliefs, family influence)
2. Part Two Questions: Factors of the health system outside the management of human resources and medical education: 2.1. Population: Population size, gender distribution, average age of the population, demographic groups (ethnic and tribal) 2.2. Health and needs of the population: Levels of self-care and health in the population, burden of diseases, severity of diseases, types of diseases 2.3. Health service delivery system: 2.3.1. Types of sectors and natures of health service delivery in terms of private, public, and academic settings 2.3.2. Performance of the health service delivery system: Levels of satisfaction, efficiency, and access 2.3.3. Levels of health service delivery: 2.3.4. Information systems and technologies: Information system, health records, technologies and innovations such as telemedicine and artificial intelligence 2.4. Financing: Insurance coverage, various revenue sources in the public and private sectors, financial resources 2.5. Facilities and equipment: Availability of hospitals and various centers, diagnostic and therapeutic equipment
3. Part Three Questions: Factors related to the human resource management system in health and medical education: 3.1. Educational system and training of general practitioners 3.1.1. What is the impact of specialization in the health sector on the demand for general practitioners? 3.1.2. Student admissions, registration 3.1.3. Evaluation and examinations: international standards 3.1.4. Characteristics of the program: duration of the program 3.2. Human resource management system: workforce (demand and supply) 3.2.1. Gender and age of physicians, students 3.2.2. What percentage of human resources and physicians are active? (Inactive, work permit holders, non-employees, full-time, retention rates in underprivileged areas) 3.2.3. Market: rates of entry into the health sector, rates of exit, retirement rates, migration rates 3.2.4. Levels of excellence and growth of human resources: levels of competence, training and empowerment courses, career paths, roles (clinical, educational, research), motivation and skills 3.2.5. Specialties for continued career paths: such as surgery, etc.
